# Retraction Note: Impact of domestic violence against pregnant women in Minia governorate, Egypt: a cross sectional study

**DOI:** 10.1186/s12884-025-08392-4

**Published:** 2025-10-27

**Authors:** Reham Elkhateeb, Ayman Abdelmeged, Samar Ahmad, Ahmad Mahran, Walaa Yehia Abdelzaher, Nermeen N. Welson, Yahea Al-Zahrani, Ahmed Mohammed Alhuwaydi, Haitham Ahmed Bahaa

**Affiliations:** 1 Department of Obstetrics and Gynecology, Faculty of Medicine, Minia University, Minia, 61511 Egypt; 2 Department of Pharmacology, Faculty of medicine, Minia university, Minia, Egypt; 3 Department of Forensic medicine and clinical toxicology, Faculty of Medicine, Beni- Suef University, Beni-Suef, 62511 Egypt; 4 Department of Internal Medicine, College of Medicine, Taif University, Taif, Saudi Arabia; 5 Saudi Board in Psychiatry, assistant professor at College of Medicine, Jouf University, Sakaka, Kingdom of Saudi Arabia

**Retraction Note: BMC Pregnancy and Childbirth 21, 535 (2021)**

**https://doi.org/10.1186/s12884-021-03953-9**

The Editor has retracted this article. Following publication, concerns were raised regarding the feasibility and results of the study reported in this article. During the investigation of these concerns, it was identified that, contrary to the statement provided in the article, the study appears not to have been approved by the Institutional ethical committee of the Faculty of Medicine, Minia University. The authors have been unable to provide satisfactory evidence to demonstrate that this study was conducted with the appropriate ethical oversight, and the Editor has therefore lost confidence in the study.

Author Nermeen N. Welson has stated that all authors disagree with this retraction.

